# Combined Use of Univariate and Multivariate Approaches to Detect Selection Signatures Associated with Milk or Meat Production in Cattle

**DOI:** 10.3390/genes15121516

**Published:** 2024-11-26

**Authors:** Michele Congiu, Alberto Cesarani, Laura Falchi, Nicolò Pietro Paolo Macciotta, Corrado Dimauro

**Affiliations:** 1 Dipartimento di Agraria, Università degli Studi di Sassari, 07100 Sassari, Italy; mcongiu1@uniss.it (M.C.); lfalchi1@uniss.it (L.F.); macciott@uniss.it (N.P.P.M.); dimauro@uniss.it (C.D.); 2 Animal and Dairy Science Department, University of Georgia, Athens, GA 30602, USA

**Keywords:** selection signatures, discriminant analysis, wright fixation index, multivariate statistics

## Abstract

Objectives: The aim of this study was to investigate the genomic structure of the cattle breeds selected for meat and milk production and to identify selection signatures between them. Methods: A total of 391 animals genotyped at 41,258 SNPs and belonging to nine breeds were considered: Angus (N = 62), Charolais (46), Hereford (31), Limousin (44), and Piedmontese (24), clustered in the Meat group, and Brown Swiss (42), Holstein (63), Jersey (49), and Montbéliarde (30), clustered in the Milk group. The population stratification was analyzed by principal component analysis (PCA), whereas selection signatures were identified by univariate (Wright fixation index, F_ST_) and multivariate (canonical discriminant analysis, CDA) approaches. Markers with F_ST_ values larger than three standard deviations from the chromosomal mean were considered interesting. Attention was focused on markers selected by both techniques. Results: A total of 10 SNPs located on seven different chromosomes (7, 10, 14, 16, 17, 18, and 24) were identified. Close to these SNPs (±250 kb), 165 QTL and 51 genes were found. The QTL were grouped in 45 different terms, of which three were significant (Bonferroni correction < 0.05): milk fat content, tenderness score, and length of productive life. Moreover, genes mainly associated with milk production, immunity and environmental adaptation, and reproduction were mapped close to the common SNPs. Conclusions: The results of the present study suggest that the combined use of univariate and multivariate approaches can help to better identify selection signatures due to directional selection.

## 1. Introduction

In recent decades, the use of genomic information derived from new technologies led to huge improvements in both the quantity and quality of milk and meat production [1]. In particular, the inclusion of BeadChip mapping of single-nucleotide polymorphisms (SNPs) has accelerated the selection progress compared to previous methodologies based only on pedigree and phenotype information [2,3]. The inclusion of genomic data (i.e., SNP panels) allowed the move from genetic selection to the genomic selection era, which shortened the generation interval [4], increased the accuracy of breeding values [5], and improved the reliability of the studies on selection signatures [6]. The intense natural and artificial selection pressure that livestock breeds have undergone left footprints of selection in the genome that are usually called selection signatures [7,8]. The analysis of these selection signatures is a way to find genomic regions involved in production and reproduction traits of interest [7]. Moreover, the identification of these footprints is useful to observe what was changed during the selection process [9] and makes it possible to find differences between selected or unselected breeds [10]. Several methods have been developed to analyze signatures of selection [9]. One of the most common is the fixation index (F_ST_), firstly proposed by Wright [11] and then refined by several authors, e.g., [9]. This statistical method is commonly used to compare breeds and helps in studying differences among populations [12,13,14,15]. F_ST_ ranges between 0 and 1, and gives an estimate of both the gene flow and the genetic distance between populations [16]. This index analyzes the genome of livestock from a univariate point of view, i.e., one SNP at a time. Genomic data, however, can be considered a multivariate system in which the variables (the genotypes and/or the SNPs) are highly correlated with one another [17]. Thus, a multivariate technique that accounts for correlations among variables and considers them simultaneously could be more profitable in analyzing such data [18,19]. On the contrary, multivariate techniques are usually more complex, require stronger assumptions, and need more computational resources than univariate approaches [20]. Several studies have analyzed selection signatures using a multivariate approach, such as stepwise discriminant analysis (SDA) [21], principal component analysis (PCA) [22], or canonical discriminant analysis (CDA) [23]. Therefore, the use of these techniques could help to identify associations between genes and economic traits such as milk production [24], meat quality [25], health, and environmental adaptation [26].

However, most of the available literature on genomic differences between beef and dairy cattle used univariate approaches, while fewer studies involved multivariate approaches [23]. However, the simultaneous use of different techniques helps to increase the reliability of selection signature studies. Investigating only signals found by more than one approach has been proposed as a valid strategy to decrease the detection of false positives [27,28,29,30].

The primary aims of this work were: (i) to study the genomic background of different cattle breeds selected for meat and milk production; (ii) to search for selection signatures associated with these economically important traits; and (iii) to compare univariate and multivariate approaches to increase the detection power of selection signatures.

## 2. Materials and Methods

Animal care and use committee approval was not needed, as data were obtained from preexisting databases.

### 2.1. Data

The datasets used in this study were retrieved using the WIDDE online database [31]. A total of 9 cattle populations were considered:

- Angus (ANG) = 62 animals [32];
- Brown Swiss (BSW) = 18 [33] and 24 [32] animals;
- Charolais (CHA) = 20 [33] and 26 [32] animals;
- Hereford (HER) = 31 animals [32];
- Holstein (HOL) = 63 animals [32];
- Jersey (JER) = 21 [33] and 28 [32] animals;
- Limousin (LIM) = 44 animals [32];
- Montbéliarde (MON) = 30 animals [33];
- Piedmontese (PIE) = 24 animals [32].

All animals were genotyped with Illumina BovineSNP50v1, and with the WIDDE tool, a total of 50,463 autosomal SNPs in common among the populations were considered. The raw dataset was submitted to quality control using PLINK 1.9 [34]. The animal and SNP call rates had to be larger than 0.95, and minor allele frequency had to be ≥0.05. Moreover, SNPs that deviated from the Hardy–Weinberg equilibrium (*p* < 10^−6^) or were not mapped according to the considered release were also discarded. After quality control, all 391 animals and 41,258 markers mapped on the 29 Bos taurus autosome (BTA) were retained for further analyses. Data were then divided in two groups: Meat (ANG, CHA, HFD, LMS, and PMT), with a total of 207 animals, and Milk (BSW, HOL, JER, and MON), with a total of 184 animals. Table 1 summarizes the dataset.

### 2.2. Population Stratification

The genomic relationship matrix (GRM) was built using the GCTA v. 1.92 [35]. In order to analyze the population stratification and graphically visualize relationships among animals, principal component analysis (PCA) was carried out on the GRM using R software v. 4.2.1 [36].

### 2.3. Selection Signature Detection

The selection signatures between the two groups, Meat and Milk, were studied using both the univariate and multivariate approaches.

The univariate approach used was the Wright fixation index (F_ST_), computed using PLINK v. 1.9 [34] and the equation proposed by Weir and Cockerham [12]. In order to remove background noise and to improve the clarity of the peaks, the estimated F_ST_ values were analyzed with LOWESS, a local weighted regression technique [37] that fits a smooth curve through points in a scatterplot. The regression was fitted using a window of 20 SNPs. Markers with a smoothed value exceeding three standard deviations from the chromosomal mean were considered interesting. A total of 20 pairwise F_ST_ comparisons were carried out: between the Meat and Milk groups and between the breeds in the Meat and Milk groups.

For the multivariate approach, the canonical discriminant analysis (CDA) was used to discover differences between the Meat and Milk groups. CDA is a multivariate technique to detect differences between groups and to study the relationships between the variables involved. CDA computes a new set of variables that are linear combinations of the original variables. The structure of these new variables, called canonical function (CAN), can be represented by the following equation:

CAN = C1X1 + C2X2+ …… + CnXn(1)

where Xi are the original variables (SNPs in this study), and Ci are the canonical coefficients. In general, if p is the number of groups, CDA extracts p-1 CANs. In this study, we tested two main groups (Meat vs. Milk), and within each group, pairwise comparisons between breeds were performed. Thus, only one CAN for each comparison was extracted.

Separation between groups was evaluated using the Mahalanobis distance between the group centroids and tested with Hotelling’s T-squared test [38]. CDA, however, can be computed only if the data matrix is at full rank, i.e., the variables are linearly independent, and the number of columns is greater than the number of rows. With genomic data, this setup is difficult to achieve, the number on variables (41,258 SNPs in the present research) being much bigger than the number of rows (391 genotyped animals in the present research). To reduce the dimension of the data matrix, stepwise discriminant analysis (SDA) was applied. This is a statistical technique specifically conceived to select a reduced subset of variables that better separate groups. With this aim, SDA was first applied within each chromosome and the retained SNPs were joined to obtain a reduced, genome-wide dataset. The last set of data was submitted to a new run of SDA until the number of linearly independent markers was lower than the number of animals involved. Finally, CDA, developed using the last selected SNPs, was exploited to test differences between breeds. Both SDA and CDA were performed using SAS software.

### 2.4. Marker-of-Interest Selection

We focused on the markers found in common among the selected SNPs independently using the two techniques (F_ST_ and CDA) to analyze differences between Meat and Milk groups. Moreover, to select only SNPs potentially associated with divergent selection signatures of these two traits, milk and meat, we removed SNPs selected in the series of pairwise comparisons between breeds within each group.

### 2.5. Gene and Quantitative Trait Loci Research

We used the R package GALLO (Genomic Annotation in Livestock for Positional Candidate Loci) [39], to search for quantitative trait loci (QTL) and to perform enrichment analysis. In order to be more conservative, the Bonferroni correction for *p*-values was applied in the enrichment analysis. As per Manca et al. [18], we used a window of 250 kb before and after each selected SNP. The annotated genes close to the SNPs were obtained from the Genome Data Viewer provided by the National Center for Biotechnology Information (NCBI). Potential phenotypes associated with each annotated gene were investigated through a comprehensive literature search. The list of mapped genes was also analyzed using STRING 12.0 (https://string-db.org, accessed on 1 August 2024).

## 3. Results

**Figure 1:**
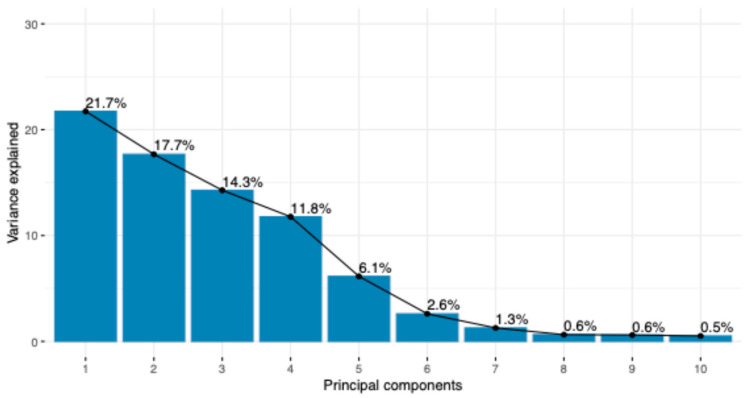
Scree plot of the variance associated with the first ten principal components extracted from the genomic relationship matrix.

The first ten principal components extracted from the GRM explained more than 77% of the total variability (Figure 1).

**Figure 2:**
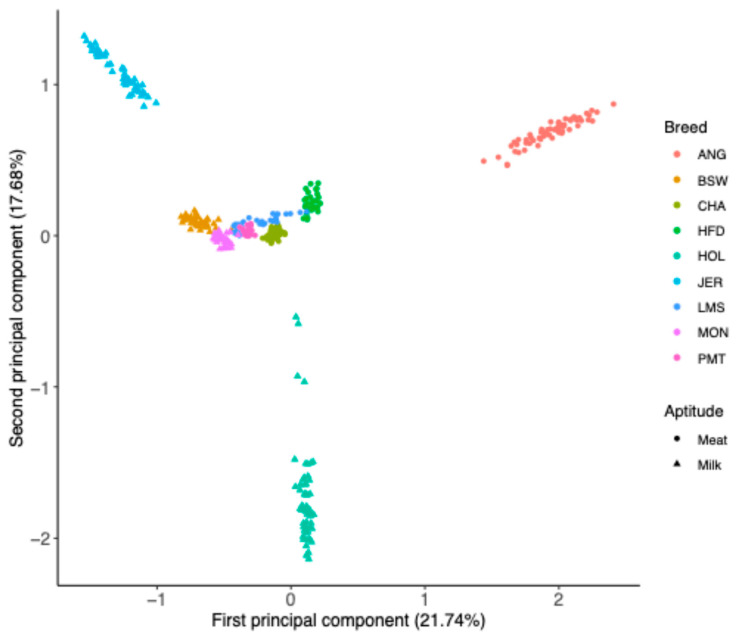
Graphical representation of the first two principal components extracted from the genomic relationship matrix.

Figure 2 displays the scatterplot of the first two principal components. Animals belonging to the Milk group (triangles in Figure 2) seem to be more shifted to the left of the graph (i.e., at negative values of PC1) compared to the Meat group (points in Figure 2). In fact, the average PC1 score computed for Milk (−0.54) was significantly lower (*p* < 0.001) than the value computed for Meat (0.48). The three more distant breeds were JER (negative values of PC1 and positive value of PC2), HOL (values close to zero for PC1 and negative for PC2), and ANG (positive values for both PC1 and PC2). All the other breeds were closely grouped around zero for the first two PCs (Figure 2).

Supplementary Table S1 shows the results of the pairwise comparisons obtained both with the univariate (i.e., F_ST_) and the multivariate (i.e., CDA) approach, applied between breeds within the Meat and Milk groups. In the Meat group, the number of interesting SNPs from F_ST_ comparisons ranged from 412 (ANG vs. HFD) to 648 (LIM vs. PMT), whereas in the Milk group, the fewest SNPs (334) were found in the comparison HOL vs. JER and the most (442) for the comparison BSW vs. JER. The number of SNPs highlighted by CDA was, as expected, lower than the number of involved animals. The lowest values were observed for ANG vs. PMT (29) and JER vs. MON (39) in the Meat and Milk groups, respectively. The largest were found for ANG vs. CHA (61) and for HOL vs. JER (60).

**Figure 3:**
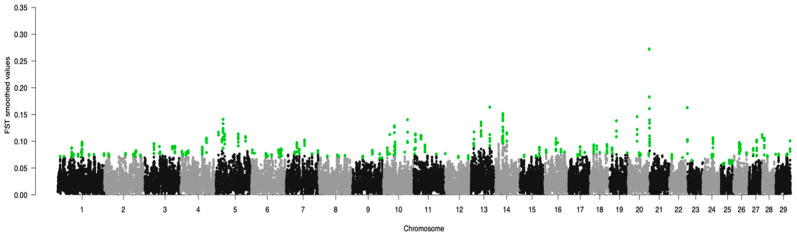
Manhattan plot of the Wright fixation index (F_ST_). Green dots represent SNPs with a smoothed value larger than three standard deviations from the chromosomic mean.

The Meat and Milk groups were differentiated by 502 and 295 markers (Table 2) found by F_ST_ (see also Figure 3) and CDA, respectively. Thirty-eight important SNPs were found on BTA1 by F_ST_ analysis, whereas the most markers (18) identified by CDA was found on BTA6 (Table 2). The fewest markers per chromosome were 6 for both CDA (chromosomes 2, 26, 28, and 29) and F_ST_ (chromosomes 12, 22, and 23).

As shown in Figure 3, the highest peak of F_ST_ was computed at the beginning of BTA21, where the marker BTB-01171128 (at 1,187,232 bp) showed the largest value (0.27). In consequence, this SNP could be considered the marker that most differentiates the two groups. Moreover, the largest average F_ST_ value (0.138 ± 0.053) was computed for BTA21, for instance, the lowest value (0.060 ± 0.003) was observed for BTA25 (Table 2).

**Figure 4:**
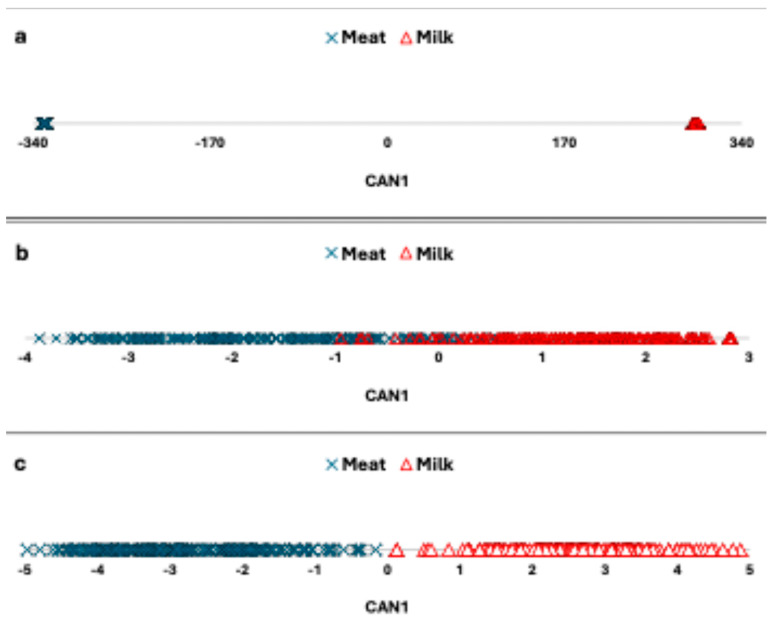
Plot of the first canonical function (CAN1) when 237 SNPs (**a**), 10 SNPs (**b**), and 18 SNPs (**c**) were used to separate the Milk (red triangles) and Meat (blue crosses) groups.

SDA selected 237 SNPs able to significantly separate (*p* < 0.0001) the Meat and Milk groups with a Mahalanobis distance between the group centroids of 391,121. Furthermore, CDA correctly assigned all animals to the two groups. As shown in Figure 4a, Meat had negative values of CAN1, whereas Milk had positive values. Since the distance between the groups was very high, animals within each group appear to be basically overlapping each other.

Based on this differentiation, SNPs with a negative CC value can be considered more associated with the Meat group and vice versa. The SNP with the largest negative value was BTA-70896-no-rs (on BTA4 at 15,692,955 bp). The largest positive value (33.83) was computed for marker ARS-BFGL-NGS-114895 located on BTA16 (at 38,205,760 bp).

Twelve SNPs were common to the lists of markers selected by F_ST_ and CDA: two of these SNPs were found in the list of markers found for the breed vs. breed pairwise comparisons within group (Supplementary Table S1), and for this reason were discarded. In consequence, attention was focused on the remaining top ten discriminant SNPs (Table 3), that were considered potentially associated with divergent selection between the Meat and Milk groups.

A new run of CDA developed using only the top 10 discriminant SNPs significantly separated (*p* < 0.001) the Meat and Milk groups with a Mahalanobis distance of 102. CDA, however, assigned animals to the correct group with a total error of 7.5% (Figure 4b). Keeping the top 10 discriminant SNPs fixed, SDA selected another 8 discriminant markers, and with these 18 SNPs, CDA was able to significantly separate the two groups and at the same time correctly assign all animals (Figure 4b).

**Figure 5:**
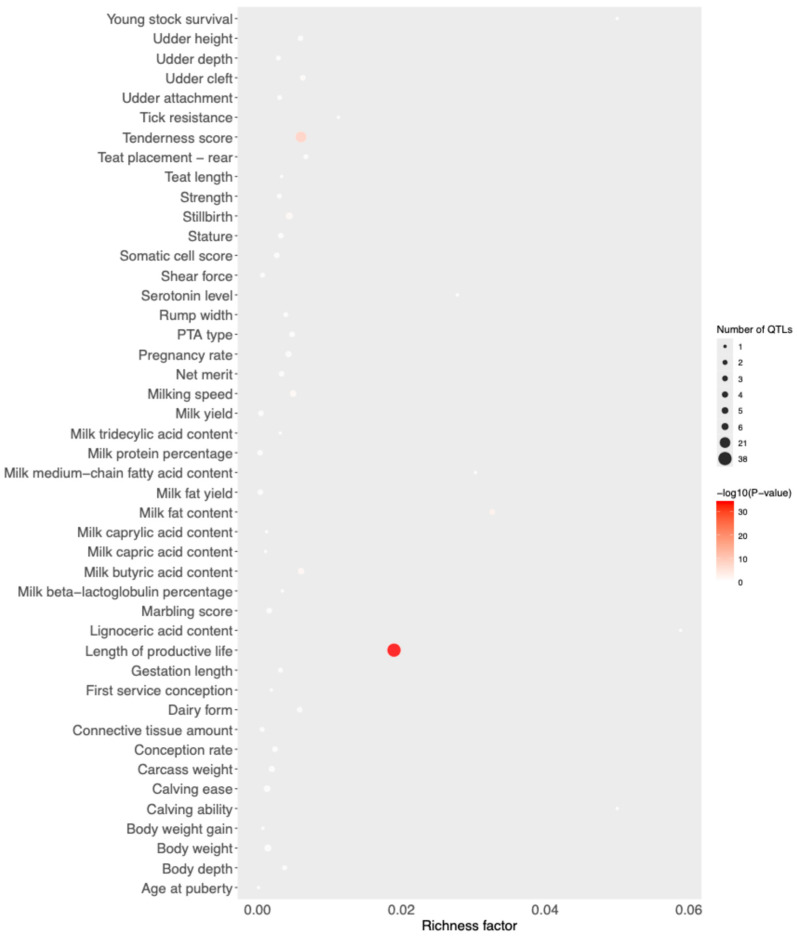
Results of the QTL enrichment analysis.

The QTL flagged by the 10 common SNPs are listed in Supplementary Table S2. The number of QTL highlighted by the Meat vs. Milk comparison was 165, clustered in 45 different terms (Supplementary Table S2) by the enrichment analysis. Three different terms (Figure 5) were significant: milk fat content (3), tenderness score (21), and length of productive life (38).

The list of all the genes mapped close (±250 kb) to the 10 common markers is reported in Supplementary Table S3. A total of 51 genes mapped on six different chromosomes were found: the most genes were found on BTA18 (22 genes) and BTA10 (18 genes), whereas only 1 gene was found on BTA7 (Supplementary Table S3). Eight genes were found in the literature to be associated with health and adaptability traits, whereas only five genes were reported to be involved in meat production (two genes on BTA10 and three on BTA18). Three genes, located on BTA18, were found in the literature to be associated with feed efficiency, and five and ten genes were reported to be related to morphology and reproduction, respectively. The largest number of genes (12) was found to related to milk production.

Based on the STRING analysis, our set of genes had significantly more interactions than expected: we found 15 edges compared to the 3 expected. A total of five GO terms, one publication, and one domain were significantly enriched (FDR < 0.05).

## 4. Discussion

The directional selection applied to improve meat and milk production has changed the phenotypic and genetic background of the current cattle breeds. In the present study, genotypes of cosmopolitan dairy and beef breeds were analyzed to investigate the genomic population stratification and to search for genomic regions associated with divergent selection. As mentioned above, data were analyzed with both a multivariate, CDA, which accounts for multiple correlations among markers, and a univariate approach, F_ST_, which analyzes markers separately. The use of two techniques at the same time can help to decrease false-positive signals and improve detection power [40]. Several studies investigated selection signatures using two or more techniques at the same time in cattle [41,42] and other species [43,44], and some of them also involved multivariate and univariate approaches [40,45].

The use of PCA on the genomic relationship matrix was not able to clearly point to differences between the Meat and Milk groups (Figure 2), even though the values of PC1 were significantly different between the two groups. Although principal component analysis is often used in the literature to detect differences among groups [46,47,48], CDA outperforms PCA in analyzing dissimilarities among groups because it is specifically intended for this purpose [49,50].

CDA significantly separated the Meat and Milk groups using 237 SNPs previously selected by SDA. The extracted canonical function correctly assigned involved animals to groups. However, we focused our attention only on the 10 markers found by the two statistical approaches that were not selected in the pairwise comparisons within the two groups. In consequence, these 10 top discriminant markers should separate the two groups only because one group was specialized to produce meat and the other group specialized in the production of milk. The 10 SNPs in fact were enough for CDA to significantly separate the Meat group from the Milk group (*p*-value < 0.001), but not all animals were assigned to the correct group, as displayed in Figure 4b, where the two groups partially overlap. The significant separation between the groups using only 10 SNPs was quite surprising. In the literature, usually a larger number of markers is reported as needed to significantly separate groups using CDA to analyze genomic differences [23,51]. The top 10 discriminant SNPs significantly captured the differences between the two groups, but were not enough to completely represent all the animals. However, after adding other eight markers, selected in a new run of SDA, all animals were perfectly identified and assigned to the correct group (Figure 4c). These results confirm the high discriminant power of the 10 selected SNPs. Two markers (ARS-BFGL-NGS-112081 and ARS-BFGL-NGS-34863) found in BTA10, with negative canonical coefficient scores, can be considered associated with meat, whereas the remaining eight markers can be considered associated with milk. The greater number of significant markers associated with milk than meat production should be not surprising, because the dairy cattle industry was more affected by genetic selection. Indeed, dairy producers used more artificial insemination than beef producers, reflected in stronger selection pressure [52]. Because of the different breeding structures, economic reasons, and breed consistency, genomic selection has been adopted more in dairy than beef cattle [53].

Three QTL terms within three different categories were found to be significant: length of productive life, tenderness score, and milk fat content. Milk fat is the most variable component in milk [54], and differences exist between dairy and beef breeds. While the former has been heavily selected for improving the quantity and quality of milk, milk production in the latter group has also been indirectly selected. In fact, milk production is the greatest single factor affecting preweaning calf weight gain, which in turn is associated with birth weight. According to Rutledge et al. [55], dam milk yield is directly responsible for 60% of the calf weight. Moreover, a direct association between maternal weaning weight and milk yield has been reported [56]. Birth and weaning weights are two of the most important traits in beef cattle [57], and the choice to improve these could also have changed the milk production and milk energy content in beef cattle breeds.

The second significant QTL term was the tenderness score, which falls within the “meat and carcass” category. Tenderness is one of the most important traits of meat quality influencing consumers’ decisions [58]. Warner–Bratzler shear force is the most common method to evaluate the tenderness of meat [59]. It is a protocol to measure force to shear across the muscle following certain parameters [60]. Although the principal factor that affects the tenderness of meat is postmortem events, other aspects can influence this trait, such as nutritional and genetic factors [61]. Meat quality is a multifactorial trait in which several genes are involved in different biological functions that influence meat traits such as marbling, tenderness, and drip loss [62]. In a meta-analysis, Berry et al. [63] found a median heritability of this trait around 0.23 [63]. Tenderness is highly correlated with traits such as marbling score, which is widely used in selection programs in beef cattle [64]. The selection of marbling score in some specialized breeds to improve tenderness was due to the strong correlation between the two traits [65]. Moravčíková et al. [66] in a selection signature study involving six beef breeds (Aberdeen Angus, Hereford, Limousin, Charolais, Piedmontese, and Romagnola) reporting common signals on regions associated with the tenderness trait, though some genes associated with tenderness were identified in an analysis of positive selection in Angus cattle [62].

The last significant QTL category was length of productive life (within the production category), which is among the most important functional traits for livestock [67] because it represents an essential indicator of animal health and welfare [68]. Longevity in dairy cattle is strongly associated with milk production (the higher the milk yield, the lower the probability of being culled), poor fertility, calf mortality, and difficult calving [69]. According to De Vries and Marcondes [70], the average productive lifespan of dairy cattle in the US is less than 3 years and the average annual cow cull rate is 38% ± 12%. In contrast, the average productive lifespan in beef cattle is around 7 to 10 years after first calving, with an approximative annual cull rate of 10–15%. Also in this case, the difference between longevity in dairy and beef cattle could be associated with the more intense genetic selection applied to dairy cattle [71].

The genes found in the present study were mainly related in the literature to reproduction, immunity and adaptation, and milk and meat production. In the list of common markers between F_ST_ and CDA, we found the SNP ARS-BFGL-NGS-100080 (located at 52.53 Mb on BTA18), which falls within the genomic region associated with calving difficulty by Purfield et al. [72]. Several studies suggested the presence of a causal mutation on CHR18 associated with calving traits, such as calving difficulty in dairy cattle [72,73]. Purfield et al. [72] analyzed the genomic background of direct calving difficulty in Holstein cattle, and they found two SNPs located on CHR18 (ARS-BFGL-NGS-109285 and BovineHD180001676) explaining 2.49% of the genetic variance in direct calving difficulty. Some other genes found in the present study were already associated with reproduction performance in the literature (Supplementary Table S2). The *DLL4* gene, located in a genomic region on BTA10 strongly associated with pregnancy loss, is involved in the development of placenta and fetal growth [74]. The *NUSAP1* gene plays a role in cell division, and its suppression can cause mitotic defects and interference with normal cell cycle progression [75]. *RAD51* is a candidate gene for discrimination of immature oocytes in relation to the age of the donor [76]. The *MIR125A* gene, located on BTA18, plays a role in the bovine preimplantation stage [77] and is strongly associated with calving problems [72]. The *ZFYVE19* gene is reported to be related to udder height in dairy cows [78], whereas the *SPINT1* gene is involved in epithelial cell differentiation and development in dairy cows [79]. Three genes (*LIM2*, *SPACA6*, and *ZNF613*) were reported to be associated with gestation length by Raschia et al. [80]. Finally, the *C18H19orf84* gene was found to be involved in spermatogenesis in Angus bulls [81].

Among the genes related to immunity and adaptation, the *GORAB* gene was reported as being involved in bovine tuberculosis protection/susceptibility by Blanco et al. [82]. The *FER* gene, mapped on chromosome 7, was found to be immunorelated in Jersey cattle [83]. It was listed among the candidate genes for selection signatures for environmental stress in African cattle [84], and it was found be involved in the regulation of innate immune response [85,86]. Other genes found in the present study related to immunity and adaptation in literature were *DNAJC17* (heat tolerance in Zebu cattle) [87], *GCHFR* [88], *MDGA2* (heat stress in cows) [89,90], *FOXF1* [91,92], *NKG7* (bovine tuberculosis response) [93], and *SIGLEC10* [94].

We found more genes related to milk than meat production (Supplementary Table S2). The *CHAC1* gene is associated with weight gain and feed intake [95]. The *CHP1* gene is listed as a candidate gene for fat deposition in sheep [96]. The *FOXC2* gene, located on BTA18, was reported as a candidate gene for meat production traits [97]. Moreover, a possible association between this gene and greater development of muscle with low fat content (i.e., double muscle) in cattle was found by Hocquette et al. [98]. The *VSIG10L* gene was associated with residual feed intake [99] and metabolic body weight [100]. The *MIR99B* was also reported as being associated with residual feed intake [101] and foot and leg conformation by Vargas et al. [102]. In the same study, the *MIRLET7E* gene was also associated with foot and leg conformation. Among the genes associated in the literature with milk production, several genes were reported to be related to fatty acid content. Several genes found in this study were associated with the fatty acid profile in Belgian Blue (*MIB1*, *MGC133647*, *ABHD3*, *SNRPD1*, *ESCO1*, and *GREB1L*) by Atashi et al. [103]. The *EXD1* gene has been reported to be associated with rear udder height [78] and milk yield in Holstein cattle by Carvalheira et al. [104]. In the latter study, a potential association between milk yield and some genes also found in the present study (*DLL4*, *CHAC1*, and *NUSAP1*) was reported.

When the genes were analyzed together using the STRING tool, we found some associations. Six genes (*LIM2*, *ETFB*, *ZNF613*, *ZNF175*, *SPACA6*, and *NKG7*) were significantly enriched in a publication about the European wild boar genome [105]. Three genes (*NKG7*, *LIM2*, and *CLDND2*) were significantly (FDR < 0.05) clustered into the protein domain (Pfam) named the “PMP-22/EMP/MP20/Claudin family”. Five different biological processes were significantly (FDR < 0.05) enriched: morphogenesis of an epithelium (GO:0002009 with *FOXF1*, *MIB1*, *GREB1L*, *DLL4*, *FOXC2*, *SPINT1*, and *GORAB* genes), tissue morphogenesis (GO:0048729 with *FOXF1*, *MIB1*, *GREB1L*, *DLL4*, *FOXC2*, *SPINT1*, *FOXL1*, and *GORAB* genes), artery morphogenesis (GO:0048844 with *FOXF1*, *PRRX1*, *DLL4*, and *FOXC2* genes), epithelial tube morphogenesis (GO:0060562 with *FOXF1*, *MIB1*, *GREB1L*, *DLL4*, *FOXC2*, and *SPINT1* genes), and morphogenesis of a branching epithelium (GO:0061138 with *FOXF1*, *GREB1L*, *DLL4*, *FOXC2*, and *SPINT1* genes).

## 5. Conclusions

Genotypes of different cattle breeds selected for milk and meat production were compared using univariate and multivariate approaches. A simple analysis of the population stratification did not highlight clear differentiation between the two groups. However, the combined use of F_ST_ and CDA allowed us to find a small number of discriminant markers between milk and meat production. Close to these SNPs, we found QTL associated with tenderness, milk fat content, and length of productive life, which are traits that differentiate between dairy and beef animals.

## Figures and Tables

**Table 1:** Summary of the dataset.

Group	Breed	Code	Animals
Meat	Angus	ANG	62
Meat	Charolais	CHA	46
Meat	Hereford	HFD	31
Meat	Limousin	LMS	44
Meat	Piedmontese	PMT	24
Milk	Brown Swiss	BSW	42
Milk	Holstein	HOL	63
Milk	Jersey	JER	49
Milk	Montbéliarde	MON	30

**Table 2:** Results of Wright fixation index (F_ST_) and canonical discriminant analysis (CDA) in separating Milk and Meat groups.

Chromosome	Average F_ST_ Value	SNP F_ST_	Average CDA Value	SNP CDA	Common SNPs
1	0.079 ± 0.008	38	−1.67 ± 8.21	12	–
2	0.076 ± 0.003	24	0.72 ± 3.72	6	–
3	0.086 ± 0.006	19	−1.76 ± 3.78	10	–
4	0.084 ± 0.009	29	−0.13 ± 9.39	16	–
5	0.109 ± 0.011	31	0.91 ± 8.36	7	–
6	0.078 ± 0.005	30	1.40 ± 7.73	18	–
7	0.086 ± 0.008	24	−1.58 ± 6.92	14	1
8	0.073 ± 0.001	9	1.26 ± 7.63	12	–
9	0.076 ± 0.005	14	−0.38 ± 1.7	9	–
10	0.095 ± 0.018	26	0.76 ± 6.17	10	2
11	0.095 ± 0.014	23	2.09 ± 3.19	8	–
12	0.070 ± 0.002	6	−1.44 ± 5.36	8	–
13	0.114 ± 0.020	16	1.49 ± 8.99	17	–
14	0.120 ± 0.019	28	2.41 ± 5.98	9	1
15	0.077 ± 0.007	11	−0.31 ± 2.72	11	–
16	0.088 ± 0.008	17	3.19 ± 10.58	15	2
17	0.079 ± 0.006	10	2.04 ± 5.74	10	1
18	0.087 ± 0.006	19	4.47 ± 4.09	7	2
19	0.093 ± 0.020	11	1.60 ± 3.86	9	–
20	0.089 ± 0.023	14	−1.27 ± 7.41	12	–
21	0.138 ± 0.053	11	−0.04 ± 5.22	8	1
22	0.071 ± 0.003	6	0.72 ± 5.79	11	–
23	0.095 ± 0.037	6	−1.21 ± 4.04	15	–
24	0.084 ± 0.014	15	1.79 ± 5.50	8	1
25	0.060 ± 0.003	12	3.98 ± 4.97	7	–
26	0.087 ± 0.007	19	−1.52 ± 6.00	6	1
27	0.088 ± 0.011	9	1.48 ± 6.13	8	–
28	0.086 ± 0.016	15	5.68 ± 9.83	6	–
29	0.079 ± 0.009	10	−2.78 ± 6.93	6	–
Total		502		295	12

**Table 3:** List of markers found in common between interesting markers from F_ST_ and CDA applied to compare Meat and Milk groups.

BTA	SNP Name	Position	CDA Score	F_ST_ Smoothed Value
7	Hapmap53962-rs29017056	107,797,993	6.7711	0.0788
10	ARS-BFGL-NGS-112081	36,489,310	−1.1832	0.0750
10	ARS-BFGL-NGS-34863	40,333,013	−9.9492	0.0983
14	ARS-BFGL-NGS-110022	38,481,264	7.8295	0.1158
16	BTB-00639530	38,137,107	5.6524	0.0853
16	ARS-BFGL-NGS-114895	38,205,760	33.8328	0.0940
17	Hapmap44543-BTA-40914	39,220,916	14.5565	0.0821
18	Hapmap47624-BTA-44484	12,578,668	0.9083	0.0931
18	ARS-BFGL-NGS-100080	57,529,674	8.6108	0.0939
24	BTB-00886858	34,750,786	7.6451	0.0959

## Data Availability

The data that support the findings of this study are available on WIDDE at http://widde.toulouse.inra.fr/widde/, accessed on 29 October 2024. Details of the considered data are listed in the Section 2.

## Supplementary Materials

The following supporting information can be downloaded at https://www.mdpi.com/article/10.3390/genes15121516/s1. Table S1: Results of Wright fixation index (F_ST_) and canonical discriminant analysis (CDA) applied within the two identified groups (Meat and Milk). Table S2: Enrichment analysis carried out on the quantitative trait loci mapped close to the significant SNPs. The significant terms (Bonferroni-corrected *p*-value < 0.05) are highlighted in red. Table S3: List of genes mapped close (±250 kb) to the common SNPs found by CDA and F_ST_ approaches. References [106,107,108,109,110,111,112,113,114,115,116,117,118,119] are cited in the Supplementary Materials.
