# Supplementary material for: Combined Use of Univariate and Multivariate Approaches to Detect Selection Signatures Associated with Milk or Meat Production in Cattle
Source: Genes (Basel). 2024 Nov 26;15(12):1516. doi: 10.3390/genes15121516 (PMC11675734; doi:10.3390/genes15121516)
Supplement: Supplementary file 1 [file genes-15-01516-s001.zip › genes-3315728-supplementary.pdf]

Supplementary Table S1. Results of the Wright Fixation Index (F_{ST}) and the Canonical Discriminant Analysis (CDA) applied within the two identified groups (MEAT and MILK).

Group	Comparison	SNP F_{ST}	SNP CDA	Common SNP
MEAT	Angus vs Charolais	457	61	6
	Angus vs Hereford	412	46	0
	Angus vs Limousine	469	56	6
	Angus vs Piemontese	448	29	5
	Charolais vs Hereford	506	41	3
	Charolais vs Limousine	577	49	5
	Charolais vs Piemontese	583	41	4
	Hereford vs Limousine	442	43	5
	Hereford vs Piemontese	465	30	1
	LMS vs Piemontese	648	38	7
MILK	Brow Swiss vs Holstein	385	55	2
	Brow Swiss vs Jersey	442	45	6
	Brow Swiss vs Montbeliarde	408	40	2
	Holstein vs Jersey	334	60	5
	Holstein vs Montbeliarde	430	43	5
	Jersey vs Montbeliarde	359	39	1

Supplementary Table S2. Enrichment analysis carried out on the quantitative trait loci mapped close to the significant SNP. The significant terms (Bonferroni corrected pvalue < 0.05) are highlighted in red.

Category	Term	N
Exterior	Dairy form	3
	Stature	3
	Strength	2
	Teat length	1
	Teat placement - rear	2
	Udder attachment	2
	Udder cleft	3
	Udder depth	2
	Udder height	3
Health	Serotonin level	1
	Somatic cell score	3
	Tick resistance	1
Meat and Carcass	Carcass weight	4
	Connective tissue amount	2
	Lignoceric acid content	1
	Marbling score	3
	Shear force	2
	**Tenderness score**	21
Milk	Milk beta-lactoglobulin percentage	1
	Milk butyric acid content	5
	Milk capric acid content	1
	Milk caprylic acid content	1
	**Milk fat content**	3
	Milk fat yield	3
	Milk medium-chain fatty acid content	1
	Milk protein percentage	3
	Milk tridecylic acid content	1
	Milk yield	3
	Milking speed	5
Production	Body depth	2
	Body weight	6
	Body weight gain	1
	**Length of productive life**	38
	Net merit	3
	PTA type	3
	Rump width	2
	Young stock survival	1

Reproduction	Age at puberty	1
	Calving ability	1
	Calving ease	5
	Conception rate	3
	First service conception	1
	Gestation length	2
	Pregnancy rate	4
	Stillbirth	6

Supplementary Table S3. List of genes mapped close (±250 kb) to the common SNP found by CDA and F_{ST} approaches.

BTA	Gene name	Gene acronym	Candidate category trait	Reference
7	FER tyrosine kinase	FER	Immunity and adaptation	[83-86]
10	Chromosome 10 C15orf62 homolog	C10H15orf62		
10	ChaC Glutathione Specific Gamma-Glutamylcyclotransferase 1	CHAC1	Meat production	[95]
10	Calcineurin Like EF-Hand Protein 1	CHP1	Meat production	[96]
10	Delta Like Canonical Notch Ligand 4	DLL4	Reproduction	[74]
10	DnaJ Heat Shock Protein Family (Hsp40) Member C17	DNAJC17	Immunity and adaptation	[87]
10	Exonuclease 3'-5' Domain Containing 1	EXD1	Milk production	[104]
10	GTP Cyclohydrolase I Feedback Regulator	GCHFR	Immunity and adaptation	[88]
10	INO80 Complex ATPase Subunit	INO80	Milk production	[106]
10	Kinetochore Scaffold 1	KNL1		
10	Nucleolar and Spindle Associated Protein 1	NUSAP1	Reproduction	[75, 107]
10	Protein Phosphatase 1 Regulatory Inhibitor Subunit 14D	PPP1R14D		
10	RAD51 Recombinase	RAD51	Reproduction	[76]
10	Ras Homolog Family Member V	RHOV		
10	Regulator of Microtubule Dynamics 3	RMDN3		
10	Serine Peptidase Inhibitor, Kunitz Type 1	SPINT1	Reproduction	[79]
10	VPS18 Core Subunit of CORVET and HOPS Complexes	VPS18	Immunity and adaptation	[108]
10	Zinc Finger FYVE-Type Containing 19	ZFYVE19	Reproduction	[78]
10	MAM Domain Containing Glycosylphosphatidylinositol Anchor 2	MDGA2	Immunity and adaptation	[89,90]
14	Cysteine Rich Secretory Protein LCCL Domain Containing 1	CRISPLD1	Milk production	[109]
14	Hepatocyte Nuclear Factor 4 Gamma	HNF4G	Milk production	[110]
16	Golgin, RAB6 Interacting	GORAB	Immunity and adaptation	
16	Paired Related Homeobox 1	PRRX1		
18	Forkhead Box F1	FOXF1	Immunity and adaptation	[91,92,111]
18	Methenyltetrahydrofolate Synthetase Domain Containing	MTHFSD	Milk production	[112]
18	Forkhead Box C2	FOXC2	Meat production	[97,98]

18	Forkhead Box L1	FOXL1	Milk production	[113]
18	Chromosome 18 C19orf84 homolog	C18H19orf84	Reproduction	[81]
18	Claudin Domain Containing 2	CLDND2	Reproduction	[114]
18	Electron Transfer Flavoprotein Subunit Beta	ETFB	Feed efficiency	[100]
18	Hyaluronan Synthase 1	HAS1	Morphology	[102,115]
18	IgLON Family Member 5	IGLON5	Feed efficiency	[100]
18	Lens Intrinsic Membrane Protein 2	LIM2	Reproduction	[80]
18	microRNA mir-12038	MIR12038		
18	microRNA mir-125a	MIR125A		
18	microRNA mir-99b	MIR99B	Meat production<br>Morphology	[101,102]
18	microRNA let-7e	MIRLET7E	Morphology	[102]
18	Natural Killer Cell Granule Protein 7	NKG7	Immunity and adaptation	[93]
18	Sialic Acid Binding Ig Like Lectin 10	SIGLEC10	Immunity and adaptation	[94]
18	SIGLEC Family Like 1	SIGLECL1		
18	Sperm Acrosome Associated 6	SPACA6	Feed efficiency<br>Reproduction	[80,100]
18	Vomeronasal 2 Receptor 408 pseudogene	VN2R408P	Morphology	[102]
18	V-Set and Immunoglobulin Domain Containing 10 Like	VSIG10L	Meat production	[99,100]
18	Zinc Finger Protein 175	ZNF175	Morphology	[116]
18	Zinc Finger Protein 613	ZNF613	Reproduction	[80, 117,118]
24	Abhydrolase Domain Containing 3, Phospholipase	ABHD3	Milk production	[103.119]
24	Establishment of Sister Chromatid Cohesion N-Acetyltransferase 1	ESCO1	Milk production	[103]
24	GREB1 Like Retinoic Acid Receptor Coactivator	GREB1L	Milk production	[103]
24	uncharacterized protein MGC133647	MGC133647	Milk production	[103]
24	MIB E3 Ubiquitin Protein Ligase 1	MIB1	Milk production	[103]
24	Small Nuclear Ribonucleoprotein D1 Polypeptide	SNRPD1	Milk production	[103]
